# Targeting SOX18 Transcription Factor Activity by Small-Molecule Inhibitor Sm4 in Non-Small Lung Cancer Cell Lines

**DOI:** 10.3390/ijms241411316

**Published:** 2023-07-11

**Authors:** Olga Rodak, Monika Mrozowska, Agnieszka Rusak, Agnieszka Gomułkiewicz, Aleksandra Piotrowska, Mateusz Olbromski, Marzenna Podhorska-Okołów, Maciej Ugorski, Piotr Dzięgiel

**Affiliations:** 1 Division of Histology and Embryology, Department of Human Morphology and Embryology, Wroclaw Medical University, 50-368 Wroclaw, Poland; 2 Division of Ultrastructural Research, Department of Human Morphology and Embryology, Wroclaw Medical University, 50-368 Wroclaw, Poland; 3 Department of Biochemistry and Molecular Biology, Faculty of Veterinary Medicine, Wroclaw University of Environmental and Life Sciences, 50-375 Wroclaw, Poland; 4 Department of Physiotherapy, University School of Physical Education, 51-612 Wroclaw, Poland

**Keywords:** non-small lung cancer, adenocarcinoma, squamous carcinoma, transcription factors, SOX18, p21, cell cycle arrest, small-molecule inhibitor

## Abstract

The transcription factor SOX18 has been shown to play a crucial role in lung cancer progression and metastasis. In this study, we investigated the effect of Sm4, a SOX18 inhibitor, on cell cycle regulation in non-small cell lung cancer (NSCLC) cell lines LXF-289 and SK-MES-1, as well as normal human lung fibroblast cell line IMR-90. Our results demonstrated that Sm4 treatment induced cytotoxic effects on all three cell lines, with a greater effect observed in NSCLC adenocarcinoma cells. Sm4 treatment led to S-phase cell accumulation and upregulation of p21, a key regulator of the S-to-G2/M phase transition. While no significant changes in SOX7 or SOX17 protein expression were observed, Sm4 treatment resulted in a significant upregulation of SOX17 gene expression. Furthermore, our findings suggest a complex interplay between SOX18 and p21 in the context of lung cancer, with a positive correlation observed between SOX18 expression and p21 nuclear presence in clinical tissue samples obtained from lung cancer patients. These results suggest that Sm4 has the potential to disrupt the cell cycle and target cancer cell growth by modulating SOX18 activity and p21 expression. Further investigation is necessary to fully understand the relationship between SOX18 and p21 in lung cancer and to explore the therapeutic potential of SOX18 inhibition in lung cancer.

## 1. Introduction

SOX18 is a transcription factor (TF) belonging to the SOX family (sex determining region Y-related high-mobility group box), which is considered one of the key transcriptional regulators during embryogenesis [1]. The SOX family consists of over 20 proteins, including SOX18, SOX7, and SOX17, which constitute the SOXF subgroup responsible for the development of the cardiovascular system, lymphangiogenesis, and blood cell differentiation processes [2,3,4,5]. Moreover, their activity has been reported during wound healing, neovascularisation, or modulation of endothelial barrier integrity, while their expression is silenced in fully differentiated cells under physiological conditions [6,7,8,9,10]. SOXF proteins have drawn attention in cancer research due to their primarily angiogenic capabilities, and their expression levels have been found to be altered in cancer cells [11,12,13]. Furthermore, growing interest and published data have led to discoveries that SOX18 is involved in the cascades of the most important molecular pathways, such as Wnt/b-catenin, mTOR, or Notch1 signalling, regulating the majority of cancer-related processes [14,15,16,17]. The mode of action of SOX18 has been described as a molecular switch for gene expression, achieved by binding to variable protein complexes or forming variable dimers, which modulate their target selectivity and/or activity [18,19]. While all SOXF TFs contain a dimerisation domain, SOX18 has the unique capability to form homodimers, which makes it stand out from other SOXF proteins as key endothelial transcription factor [20]. Despite this body of evidence, our understanding of the mechanisms of SOX18-mediated transcriptional regulation remains scattered, as every report regarding SOX18’s contribution in carcinogenesis has focused on only a few specified proteins. Current evidence suggests that SOX18 has an oncogenic role in carcinogenesis, promoting migration, invasion, and proliferation in cell lines of various cancer origins, including bladder, breast, cervix, colon, larynx, liver, kidneys, osteosarcoma, pancreas, and prostate [14,16,21,22,23,24,25,26,27,28,29,30,31,32,33,34,35]. However, a study conducted on thyroid cancer cell lines reported that SOX18 acts as a tumour suppressor, indicating the complexity of its activity, which is likely dependent on the molecular context present in particular tumours [16]. Moreover, its unique mode of action as a homodimer may be crucial for the reported tumorigenic role of SOX18, as opposed to SOX7 and SOX17 [20]. In light of these findings, SOX18 appears to be an attractive molecular target for cancer research in studies aimed at treatment or molecular-based mechanistic investigations.

Investigating the mode of action of TFs is crucial due to their frequent differential expression in pathological conditions. In diseases, such as cancer, the survival of tumour cells requires the reprogramming of molecular network to maintain constant cell divisions during starvation or hypoxic conditions [36]. However, developing pharmacological molecules to target TF activity is very challenging since they are shielded by the nuclear membrane, and their three-dimensional structure is not fully resolved. Moreover, little is known about the complete spectrum of their interaction and resulting molecular responses [37]. Therefore, the investigation of TFs has mainly focused on gene expression modulation, such as gene silencing, knockdown, or overexpression [38,39,40]. However, recent studies have shown the potential of developing specific inhibitors for TFs. For instance, Francois group has reported the development of a specific inhibitor, small-molecule 4 (Sm4), that targets SOX18 TF [37]. Sm4 was derived from a natural product identified in brown alga extract and was found to be effective in disrupting homodimerisation as well as blocking other protein–protein interactions, resulting in the inhibition of transcriptional activity of SOX18 TF. Genomic, proteomic, and biophysical techniques were used to characterize Sm4’s properties toward the SOX TFs family, and in vitro cytotoxic and luciferase reporter assays were performed at the COS-7 normal renal fibroblast cell line [37]. The biological responses were then evaluated in vivo, using a transgenic zebrafish reporter validated as a readout of the combinatorial activity of SOX7 and SOX18 and the implantation of 4T1.2 mammary carcinoma cells into a mouse model [41]. The study found that Sm4 significantly inhibited neovascularisation, resulting in a lower metastatic rate in the Sm4-treated group. However, the tumour size was not affected by the treatment, indicating that Sm4 has no effect on mouse carcinoma cell proliferation. Despite these findings, the biological responses dependent on SOX18 are highly influenced by the specific type of cancer. As a result, the outcomes may vary in other molecular contexts.

Non-small cell lung cancer (NSCLC) is constantly listed at the top of the world’s epidemiological statistics, always presenting the highest mortality among other cancers [42]. The intricate nature of lung cancer biology hinders the effectiveness of therapeutic approaches targeting a single specific target. Although there are molecular aberrations that are frequently observed in NSCLC, targeting them alone is insufficient to fully eliminate cancerous and pre-cancerous cells from the body [43]. Additionally, NSCLC is often diagnosed in advanced or metastatic stages, and is highly metastatic, making it a significant challenge for treatment [44]. SOX18 has emerged as a potential novel target in NSCLC due to its involvement in neoangiogenesis and modulation of endothelial barrier integrity, which are important in cancer progression [13]. However, studies on SOX18 expression levels in NSCLC have yielded contradictory results. Our previous studies found variable SOX18 expression levels in NSCLC patients, with some exhibiting weak or strong expression levels [21]. Notably, increased cytoplasmic SOX18 expression appeared to be a negative prognostic marker [21]. Recently published bioinformatic reports of public proteomic databases have revealed downregulation of SOX18 in NSCLC, with strong negative correlation of SOX family proteins expression with tumour hypoxia [17]. Epigenetic studies have also shed light on the contribution of SOX18 TFs in lung tumorigenesis. High methylation of *SOX18* promoter has been reported in few studies, which partially explains the low levels of *SOX18* mRNA found in lung cancer cohorts [45,46]. However, our research group has shown that reduced levels of certain microRNAs in NSCLC in comparison to normal lung transcriptome, increase *SOX18* transcript and activate protein synthesis [47,48]. This evidence in turn, may explain the high expression of nuclear and cytoplasmic SOX18 in NSCLC samples. Despite these findings, the full range of molecular activities of SOX18 in NSCLC remains under investigation for a better understanding of its contribution, role, and mode of action in the disease. Here, we investigated the impact of Sm4 as a SOX18 inhibitor in NSCLC cell lines.

## 2. Results

### 2.1. Sm4 Treatment Shows Comparable Cytotoxicity in NSCLC and IMR-90 Cell Lines

**Figure 1:**
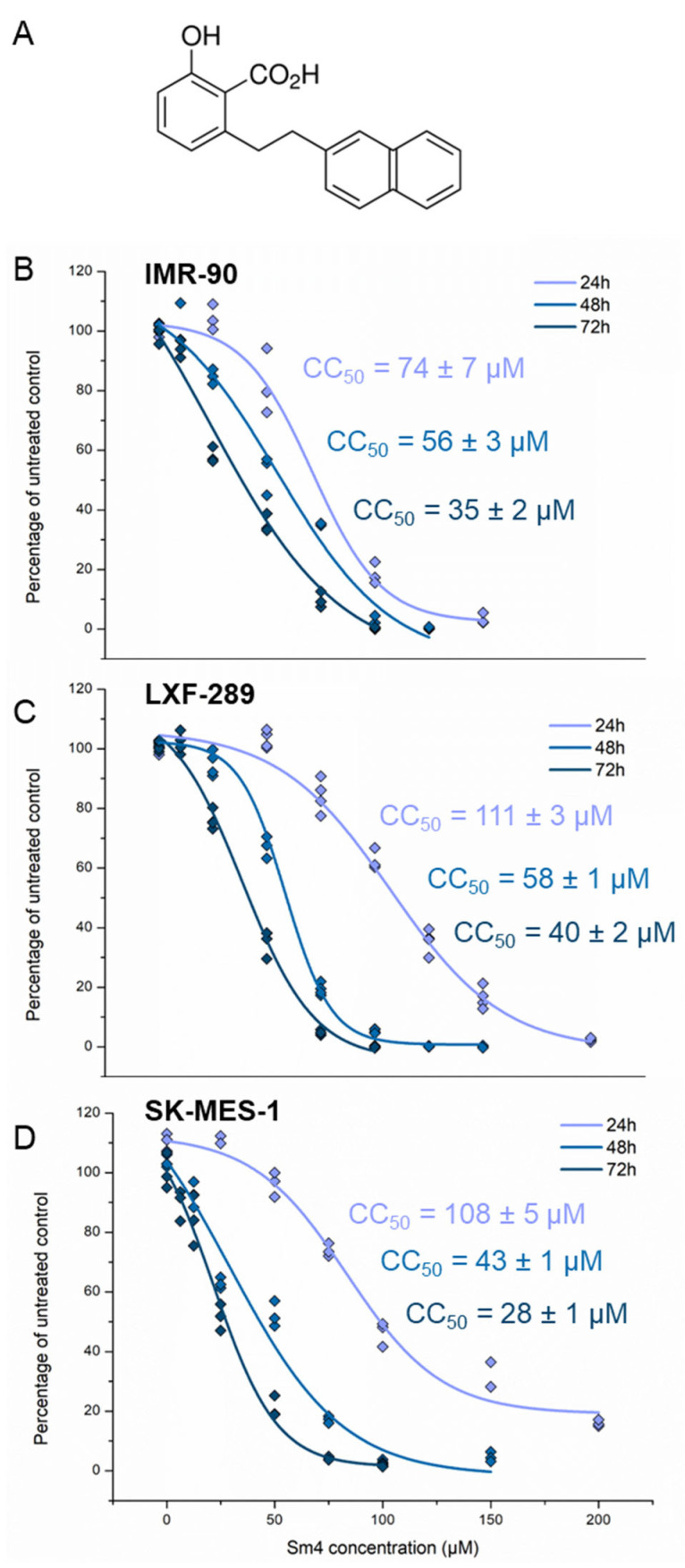
Sm4 effect on cells survival. (**A**) Sm4 chemical formula; cytotoxicity of Sm4 treatment for 24, 48, and 72 h in (**B**) lung fibroblasts IMR-90 cell line, (**C**) adenocarcinoma LXF-289 cell line, (**D**) squamous carcinoma SK-MES-1 cell line.

To determine the cytotoxic effect of Sm4 (Figure 1A) on studied lung cancer cell lines, an MTT cell assay was performed. Post-hoc analysis revealed that Sm4 cytotoxicity was time- and concentration-dependent (*p* < 0.001) in all assayed cell lines (Figure 1B–D). The normal lung fibroblast cell line (IMR-90) was more sensitive to Sm4 treatment at 24 h than the cancer cell lines. The 50% cytotoxic concentration (CC_50_) value of the fibroblast population was achieved at 74 ± 7 µM of Sm4, which was significantly lower than the values of 108 ± 5 µM and 111 ± 3 µM obtained in SK-MES-1 and LXF-289, respectively (*p* < 0.001). At longer incubation times, the IMR-90 and LXF-289 cell lines presented comparable resistance to Sm4 (*p* = 0.76). The results revealed CC_50_ values to be different between all tested cell lines at the 72 h treatment period (*p* < 0.05), from which the most sensitive appeared to be the SK-MES-1 cell line. Based on the cytotoxicity results, 10 and 20 µM Sm4 treatments were selected for subsequent experiments.

### 2.2. Disruption of SOX18 Activity Alters Transcript Levels of SOX7 and SOX17 but Not Protein Expression

The SOXF protein group is characterised by high homology among its members and is believed to have redundant functions in specific, not fully understood circumstances [13,17,49]. Therefore, our initial hypothesis was that Sm4 inhibition of SOX18 would trigger the expression of SOX17 or SOX7 proteins, leading to the activation of antiproliferative mechanisms.

**Figure 2:**
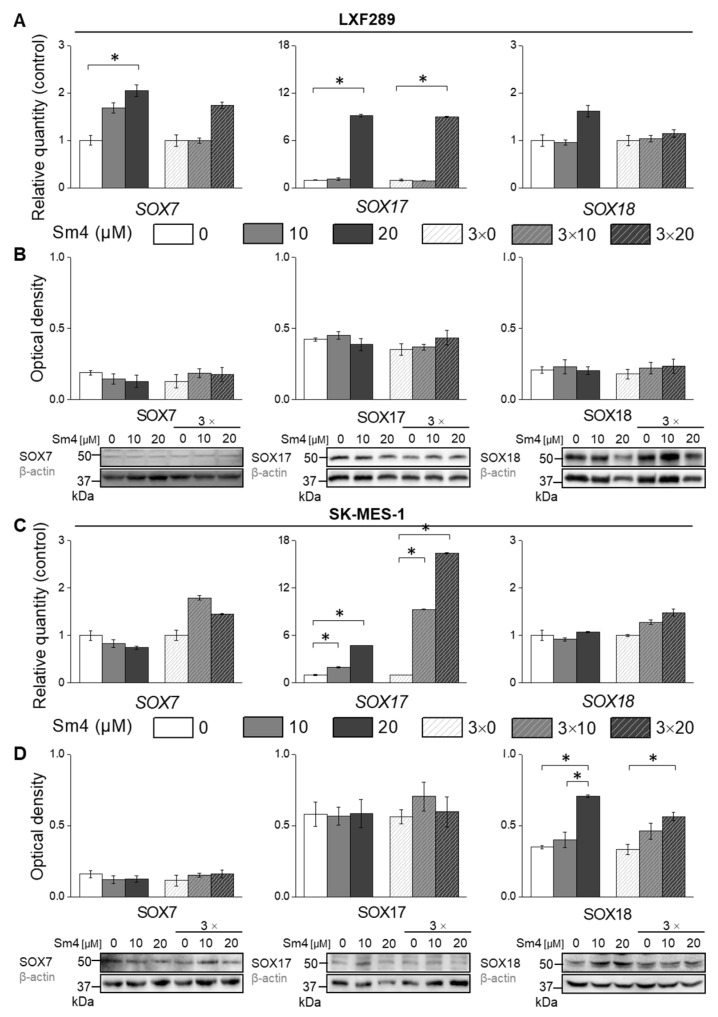
Gene expression analysis by qPCR (**A**,**D**) and protein levels by Western blot (**B**,**C**) of SOX7, SOX17, and SOX18 in LXF-289 (**A**,**B**) and SK-MES-1 cell lines (**C**,**D**). The statistical analysis of protein expression changes was based on densitometry results obtained from three independent experiments. The asterisk indicates *p* < 0.05.

In LXF-289 cells, addition of Sm4 induced the expression of *SOX7* and *SOX17* independently of the inhibitor treatment scheme, but *SOX18* was only increased upon one-time Sm4 treatment (Figure 2A). However, we did not detect an increase in protein levels (Figure 2B). In the SK-MES-1 cell line, SOX17 expression was increased upon Sm4 treatment, but this did not result in increased protein levels (Figure 2C). Interestingly, although *SOX18* transcription remained unaltered, we observed increased SOX18 protein levels (Figure 2C,D).

### 2.3. Sm4-Mediated Cell Cycle Arrest Exhibits a Prominent S-phase Cell Accumulation in LXF-289 Cell Line

**Figure 3:**
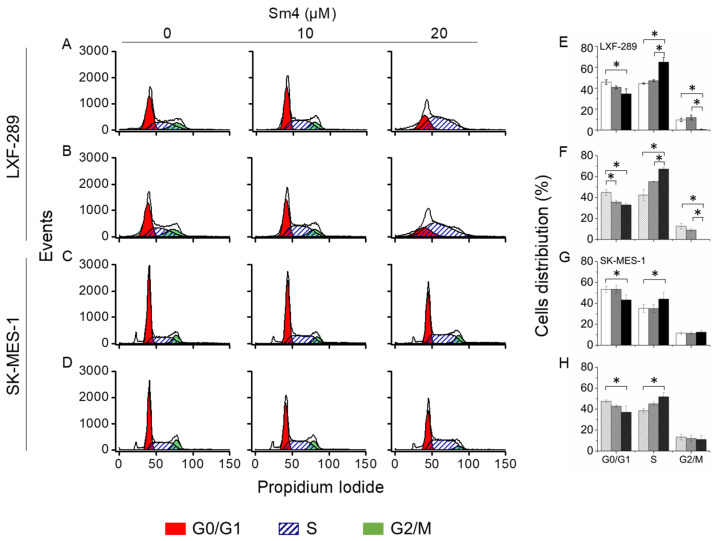
Histograms for cell cycle analysis for one- (**A**) or three-time treatment (**B**) of LXF-289 and one- (**C**) or three-time treatment (**D**) of SK-MES-1 cell lines at three Sm4 concentrations. Analysis of the cells distribution (%) are derived from the histograms (**E**–**H**). Presented results were obtained from three independent cell cycle measurements. The asterisk indicates *p* < 0.05.

To evaluate putative cell cycle disruption triggered by Sm4, cancer cell lines were treated with 10 and 20 µM of the inhibitor for 72 h. The distribution of cell populations, in particular cell division phases, was evaluated. A significant S-phase arrest was observed in the LXF-289 adenocarcinoma cell line, with a complete disappearance of the G2/M cell population in both treatment schemes by 20 µM Sm4 (Figure 3A,B,E,F). Similarly, treatment with 20 µM Sm4 increased the number of cells in the S-phase in SK-MES-1 cells, confirmed by a significant decrease in G1-phase population (Figure 3C,D,G,H). Results in both cell lines confirmed that the inhibitor showed significant effects on the progression of cell division phases at a concentration of 20 µM only. A one-time treatment of 10 µM Sm4 had no effect, while results from repeated treatment with 10 µM Sm4 presented a tendency toward S-phase arrest. Moreover, the cell cycle analysis performed in IMR-90 normal fibroblast cell line after 72 h from the Sm4 one-time exposure showed no differences between control and treated cells (Figure S1).

### 2.4. Differential Effects of Sm4 on Cyclin Expression in LXF-289 and SK-MES-1 Cell Lines

**Figure 4:**
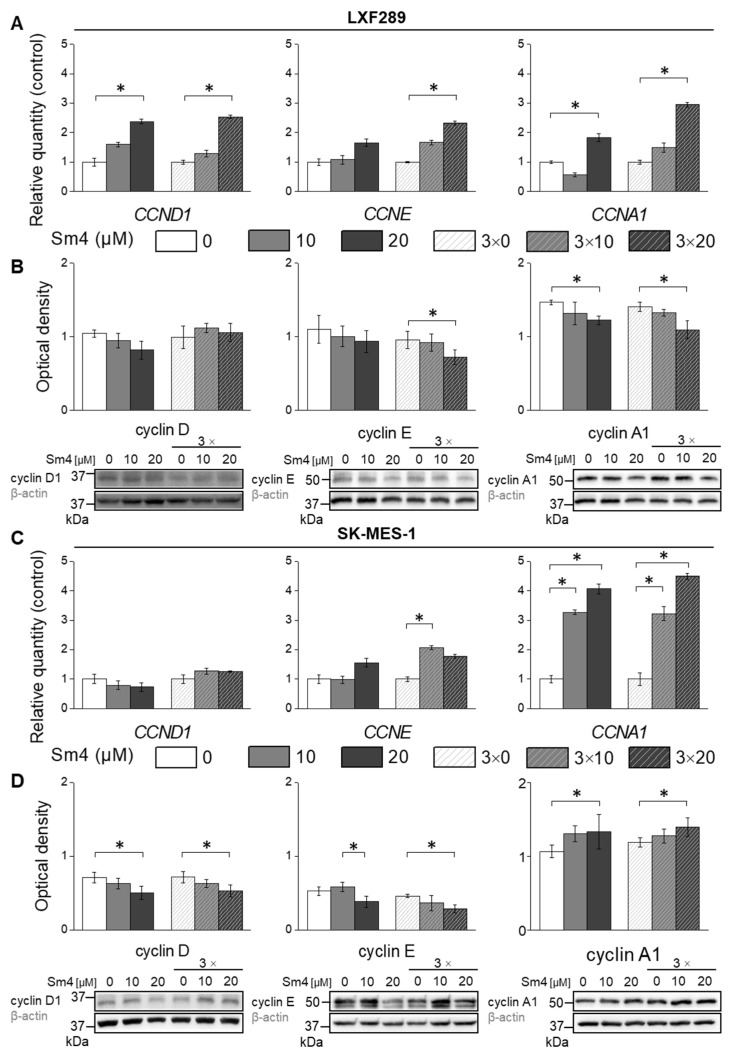
Gene expression analysis by qPCR of CCND1, CCNE, and CCNA1 and protein levels by Western blot of cyclin D, cyclin E, and cyclin A1 in LXF-289 (**A**,**B**) and SK-MES-1 cell lines (**C**,**D**). The statistical analysis of protein expression changes was based on densitometry results obtained from three independent experiments. The asterisk indicates *p* < 0.05.

The observed S-phase arrest caused alterations in the expression of proteins involved in cell cycle regulation, prompting the investigation of potentially implicated proteins due to SOX18 inhibition. As a result, the expression of cyclin D1, E, and A1 was assessed through Western blot analysis (Figure 4). In LXF-289 cells, independent of the inhibitor addition scheme, treatment with 20 µM Sm4 led to increased gene transcription of CCND1, CCNE, and CCNA1 (Figure 4A). However, despite the observed increase in gene transcription, a notable decrease in cyclin E and A1 levels was observed, as depicted in Figure 4B. Sm4 treatment resulted in the upregulation of CCNE and CCNA1 expression in the SK-MES-1 cell line, whereas CCND1 mRNA levels were not affected (Figure 4C). Furthermore, in SK-MES-1 cells, increased CCNE mRNA did not lead to a corresponding increase in protein levels (Figure 4D), while an increase in CCNA1 resulted in an increase in cyclin A1 protein levels.

### 2.5. Sm4-Induced SOX18 Activity Inhibition Upregulated p21 Expression

**Figure 5:**
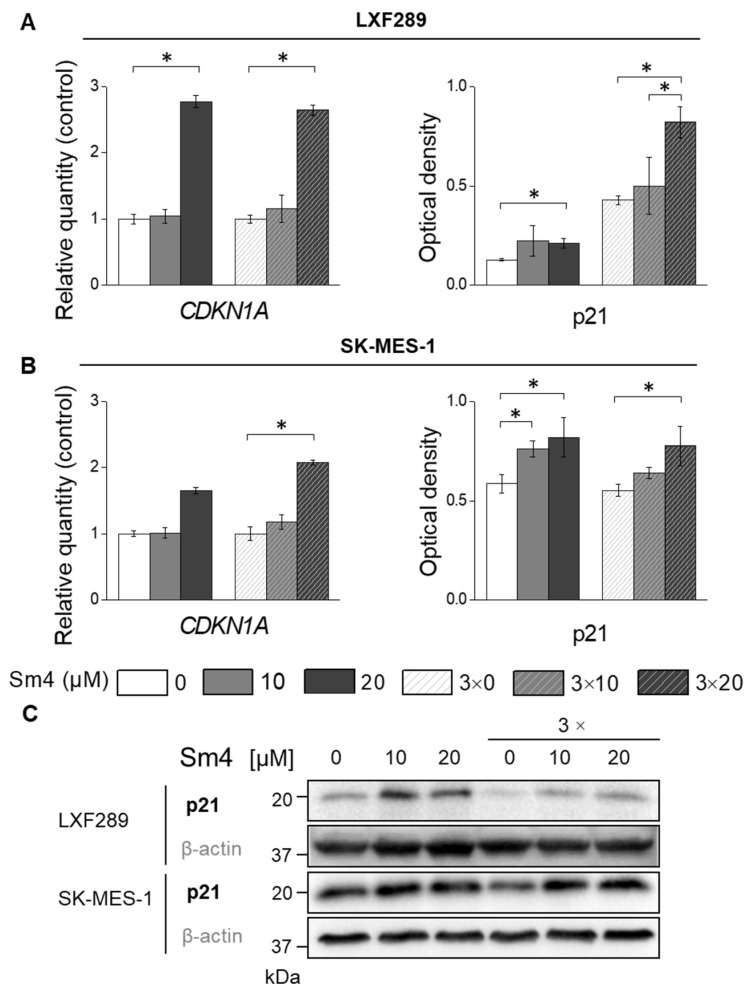
Gene expression analysis by qPCR of CDKN1A and protein levels by Western blot of p21 in LXF-289 (**A**) and SK-MES-1 cell lines (**B**). Western blot for p21 and β-actin in LXF-289 and SK-MES-1 cell lines for one- and three-time Sm4 treatment (**C**). The statistical analysis of protein expression changes was based on densitometry results obtained from three independent experiments and two technical repetitions (*n* = 6). The asterisk indicates *p* < 0.05.

After observing S-phase arrest in both cell lines, we sought to investigate the p21, a key regulator of the transition from S to G2/M phase (Figure 5). The analysis of gene ex-pression revealed a significant upregulation of CDKN1A expression, which subsequently translated into an increase in p21 protein levels upon treatment with 20 µM Sm4 in both cell lines.

### 2.6. SOX18 and p21 IHC Expression Levels Are Negatively Correlated in Lung Cancer Tissue Samples

**Figure 6:**
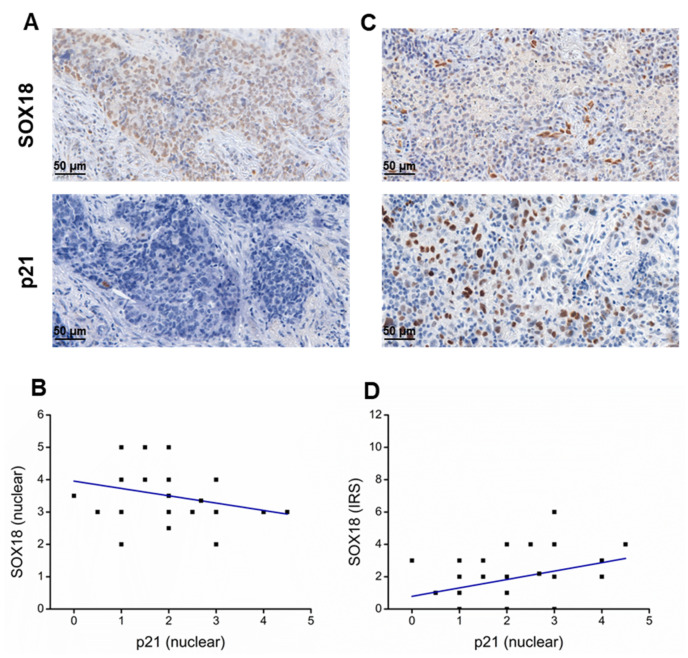
IHC of tissue samples (*n* = 48) revealed correlations between p21 and SOX18. Representative pictures of nuclear p21 and SOX18 immunodetection (original magnification ×400). (**A**) Upper figure shows high expression of SOX18, while bottom figure shows singular immunodetection of p21 in the same sample. (**B**) Negative correlation plot between nuclear expressions of SOX18 and p21 observed in lung cancer tissue samples. (**C**) Upper figure presents high immunoreactive score of Remmele and Stegner (IRS) of SOX18, and bottom figure shows correlating high expression of nuclear p21 observed in the same sample. (**D**) Positive correlation plot between nuclear expressions of IRS SOX18 and p21 observed in lung cancer tissue samples.

To check the expression patterns and intensity of SOX18 and p21 in clinical tissue samples, we performed evaluations based on IHC staining. Positive signal of SOX18 was found as nuclear or cytosolic and p21 was detected only in the nuclei (Figure 6A,C). Pearson correlation analysis of expression grading results revealed a negative correlation (*r* = −0.27) between nuclear SOX18 and nuclear p21 expression (Figure 6B), while a positive correlation (*r* = 0.33) between cytosolic SOX18 and nuclear p21 immunodetection levels (Figure 6D). Additionally, we performed a correlation analysis of *CDKN1A* and *p21* genes expression in lung using the publicly available RNA sequencing database OncoDB https://oncodb.org/ (accessed on 20 June 2023). Our analysis revealed a positive correlation between the expression of both genes, with correlation coefficients of *r* = 0.22 and *r* = 0.21 in adenocarcinoma and squamous cell lung cancer subtypes, respectively (Figure S2).

## 3. Discussion

Previous studies indicated that SOX18 exhibited oncogenic activity in various types of cancers. Jethon et al. [13] indicated that expression of SOX18 correlated with poor patient outcome in non-small cell lung cancer. Sm4 is a novel compound that was identified through extensive screening for putative SOX18 inhibitors, as presented by Fontaine et al. [37]. Their investigation of the mode of action and target selectivity of Sm4 revealed that it highly depended on concentration. Cytotoxicity assays showed that Sm4 had a CC50 ranging from 50 to 100 µM in human embryonic kidney 293 cells and Hep G2 hepatocellular carcinoma cells, and a 24 h CC50 of 117 µM in COS-7 monkey kidney fibroblast-like cell line. These results suggest that Sm4 exhibits variable cytotoxicity depending on the cell line [37]. To date, there have been no studies investigating the effect of Sm4 treatment on cancer cells in vitro; therefore, this study attempted to examine this aspect.

The sensitivity of the IMR-90 cell line to Sm4 was greater than that of the cancer cell lines after 24 h of treatment. However, over time, the IMR-90 cell line developed increasing resistance to Sm4, and after 72 h, the cytotoxic effect of Sm4 was similar in all cell lines. It is noteworthy that there was a surge in the cytotoxicity of Sm4 in both cancer cell lines after 48 h, indicating that the inhibition of SOX18 activity, which affected cell function, required more than 24 h to take effect. The results of our study suggest that Sm4, a SOX18 inhibitor, has cytotoxic effect on both NSCL cell lines, LXF-289 and SK-MES-1, and IMR-90 cell lines, which could potentially limit its therapeutic value. However, in vivo studies have evaluated the efficacy of Sm4 treatment in mice with mammary gland carcinoma and have not reported any toxicity or significant side effects [41,50]. Further research is required to better understand the mechanisms underlying the cytotoxic effects of Sm4 on lung cells and to determine its potential clinical utility.

The SOXF subgroup is composed of three closely related transcription factors that have been reported to be redundant and correlated with each other [2,12,17,51,52,53]. While SOX18 has been found to be overexpressed in tumours, SOX7 and SOX17 have been reported to be downregulated [9,12,54,55,56,57]. Furthermore, SOX7 and SOX17 have been demonstrated to possess antiproliferative functions in cancer cells [54,55,56,57,58]. Despite our initial hypothesis regarding the putative upregulation of SOX7 or SOX17 causing Sm4-mediated proliferation inhibition, our evaluation of Sm4-induced changes in gene and SOXF protein expression did not confirm this hypothesis. Although we detected a significant upregulation of *SOX17* gene expression, no changes in protein expression level were observed. These results prompted us to search for other molecular events responsible for cell cycle arrest.

The cell cycle assay revealed that LXF-289 cells underwent significant S-phase arrest, while treatment with 20 µM Sm4 increased the number of cells in S-phase and G2/M-phase in SK-MES-1. SOX18 has been studied for its role in cell cycle control, but results vary depending on the cancer type. In previous studies, *SOX18* silencing induced G0/G1 arrest in colorectal cancer, laryngeal, hepatocellular carcinoma, renal and blader cancer, while S-to-G2/M inhibition was observed in SOX18 knockdown osteosarcoma cells [14,16,21,22,23,24,25,26,27,28,29,30,31,32,33,34,35]. To shed more light on the observed disturbances of the cell cycle, we investigated the response of cyclins that controls the major events of cell cycle progression at the transcript and protein levels. The results showed downregulation of cyclin D and E, despite their increased gene expression, confirming the involvement of SOX18 in cell cycle progression. The difference in the observed cell cycle phases distribution between squamous cell carcinoma and lung and adenocarcinoma cell lines may be explained by the opposing results of cyclin A1 expression, which is responsible for the S-to-G2/M phase transition. This suggests that Sm4-mediated SOX18 inhibition can activate different pathways depending on the cell type, resulting in contradictory molecular behaviours. Our findings suggest that Sm4 has the potential to disrupt the cell cycle and target cancer cell growth.

Intriguingly, we observed an upregulation of SOX18 in SK-MES-1 cells following treatment with Sm4. This may be a response to the inhibited function of SOX18, resulting in the accumulation of not fully functional protein. Moreover, the observed upregulation could potentially provide an explanation for the relatively lower inhibitory effect of Sm4 on the cell cycle in SK-MES-1 cells compared to LXF-289 cells. In SK-MES-1 cells, a more notable accumulation in the S-phase was observed, while no significant differences in SOX18 concentration were detected between the two cell lines.

**Figure 7:**
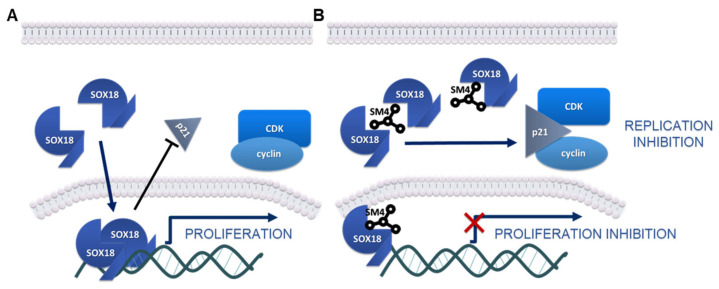
Schematic representation of the SOX18-dependent regulation of p21 expression and antiproliferative activity. (**A**) Functional SOX18 dimers control and induce the transcription of target genes that downregulate p21. (**B**) The inhibition of SOX18 homodimerisation ability mediated by Sm4 inhibits the transcription of specific genes, resulting in the upregulation of p21 and cell division arrest.

The S-to-G2/M phase transition in the cell division cycle is controlled by many proteins, among which p21 is considered one of the key regulators [59,60]. The p21 protein has been found to be upregulated in cancer cells as a molecular response to various small-molecule treatments that trigger S-phase arrest in different cancer cells [61,62,63,64,65,66]. Our results confirm significant Sm4-mediated p21 upregulation in both cancer cell lines, suggesting that modulation of SOX18 activity affects p21 expression and activity (Figure 7). The p21 protein is a low molecular weight macromolecule called a cyclin-dependent kinase inhibitor, presenting a variety of modes of action that orchestrate the progression of the cell division cycle [60,67]. Increased p21 expression and its nuclear accumulation overcome the strength of cyclin-kinases signalling by arresting the cell cycle at G1-phase or S-phase, as we have observed in our experiments (Figure 7) [68]. Zhu et al. reported the only study that has explored the relationship between SOX18 and p21 expression. Their study demonstrated that SOX18 silencing led to an increase in p21 expression, while SOX18 overexpression decreased p21 expression in osteosarcoma cells [35]. Notably, they observed G0/G1 cell cycle arrest, which, in conjunction with increased p53 expression, suggested that the inhibition of cell cycle progression might be mediated by the p21/p53 complex [35]. In contrast, our study was conducted on cell lines with p53 mutations, implying that the observed S-phase arrest might have occurred in a p53-independent manner. However, this pathway of cell cycle arrest has not been further investigated in this work. The anticancer effects of p21 include the formation of a p21/PCNA complex, which successfully hinders the replication process, providing support for our findings [67,68,69].

To reinforce our hypothesis that SOX18 may have a role in regulation of p21 expression in lung cancer cells, we performed an assessment of IHC staining in clinical tissue samples obtained from lung cancer patients. As a result, we demonstrated a correlation between SOX18 expression and p21 nuclear presence. It is important to note that the activity of a transcription factor, such as SOX18 depends on its location. Cytoplasmic staining indicates an inactive state of SOX18 [21,70]. This may explain the positive correlation between the IRS grading and p21 nuclear immunopositivity, as well as the negative correlation between active SOX18 found in nuclei and p21 in our assay. However, our analysis of *CDKN1A* and *p21* mRNA levels in lung tumours indicated a positive correlation. We hypothesise that the positive correlation may originate from epigenetic mechanism. Nevertheless, the observed correlation supports our results regarding their correlation. These findings suggest a complex interplay between SOX18 and p21 in the context of lung cancer (Figure 7). Further investigation is necessary to fully understand the relationship between these two proteins in the disease.

## 4. Materials and Methods

### 4.1. Materials

Inhibitor of transcription factor SOX18—small-molecule 4 (Sm4, Cat. SML1999) was purchased from Sigma-Aldrich (Merck KGaA, Darmstadt, Germany) and reconstituted in DMSO according to the manufacturer’s recommendations as 2.5 mg/mL.

### 4.2. Experimental Design

Following the cytotoxicity assay, two concentrations of Sm4 were chosen for further experiments. The treatments were carried out for 72 h using two protocols: (i) Adding Sm4 at the desired concentration once, 24 h after plating the cells, or (ii) replacing the media with the inhibitor at 10 and 20 µM, or vehicle every 24 h. The aim of this methodology was to investigate whether a single exposure to Sm4 could inhibit SOX18 activity for 72 h or whether repeated treatments were necessary to achieve this effect.

### 4.3. Cell Culture

Two human lung cancer cell lines of two different and most frequent subtypes, i.e., LXF-289 (adenocarcinoma) and SK-MES-1 (squamous cell carcinoma) were purchased from CLS (Cell Lines Service GmbH, Eppelheim, Germany) and normal lung fibroblast cell line was received from ATCC (Manassas, VA, USA). All cell lines were cultured in Eagle’s Minimum Essential Medium (EMEM, Lonza, Basel, Switzerland), supplemented with 10% Fetal Bovine Serum, HEPES, L-glutamine, sodium purvate, non-essential amino acids, 100 units/mL penicillin, and 100 µg/mL streptomycin (Sigma-Aldrich, Merck KGaA, Darmstadt, Germany). All cells were maintained under 95% air and 5% CO_2_ atmosphere at 37 °C in humidified incubator.

### 4.4. MTT Assay

Cytotoxicity of Sm4 was assessed using the MTT method. Cells were seeded into three 96-well plates at a concentration of 4 × 10^3^/well. After 24 h, the media was replaced with media containing Sm4 at different concentrations (ranging from 5 to 200 µM). Each plate was then incubated with the treated media for different time periods: 24 h, 48 h, and 72 h. After incubation, the culture medium was removed and 100 µL of sterile MTT solution (0.5 mg/mL, Invitrogen, Thermo Fischer Scientific, Waltham, MA, USA) was added to each well. Following 4 h of incubation at 37 °C, the MTT solution was gently removed and all formed formazan crystals were dissolved in 100 µL of DMSO (Dimethyl Sulfoxide, Sigma-Aldrich, St. Louis, MO, USA) by shaking the plate for 10 min. Absorbance was measured at 460 nm using a microplate reader (Lx800, Bio-Tek, Winooski, VT, USA). The experiments were performed in triplicates.

### 4.5. Cell Cycle Assay

After a specific incubation time with the Sm4 inhibitor, cells were harvested using Trypsin-EDTA solution (0.25%, Sigma Aldrich, St. Louis, MO, USA) and then centrifuged. The cells were washed twice with cold PBS, suspended in 2 mL of 70% ice-cold ethanol solution for fixation, and subsequently centrifuged. The cells were then washed twice with cold PBS. Thereafter, the pellets were suspended and incubated in 300 µL of propidium iodide (PI)-RNAse A solution (Sigma Aldrich; Merck KGaA, St. Louis, MO, USA) for 30 min at 37 °C in the dark. DNA content was analysed using a flow cytometer (BD Biosciences, Franklin Lakes, NJ, USA). The obtained FCS files of PI spectra were assessed using ModFit LT 5.0 software (Verity Software House, Topsham, ME, USA). The experiments were performed in triplicates.

### 4.6. Gene Expression by qPCR

Cells were harvested from culture flasks, centrifuged, and washed twice with PBS. The obtained pellets were stored at −80 °C until all materials were collected. RNA isolation was performed using RNeasy Mini Kit (Qiagen, Hilden, Germany) according to the manufacturer’s protocol. RNA was dissolved in RNA-pure water, and the concentration was measured using NanoDrop 1000 spectrophotometer (Thermo Fischer Scientific, Waltham, MA, USA). For reverse transcription, 500 ng of RNA was used, and cDNA synthesis was performed using iScript cDNA synthesis kit (Bio-Rad, Marnes-la-Coquette, France) in a C1000 Touch thermal cycler (Bio-Rad, Marnes-la-Coquette, France). The TaqMan specific probes used in the experiment were purchased from Thermo Fischer Scientific (Waltham, MA, USA). The expression of the following genes was analysed: *SOX18* (Hs00746079_s1), *SOX17* (Hs00751752_s1), *SOX7* (00846731_s1), *CCNA1* (Hs00171105_m1), *CCND1* (Hs00765553_m1), *CCNE1* (Hs01026536_m1), *CDKN1A* (Hs00355782_m1), and *SDHA* (Hs00188166_m1) as a housekeeping gene. Gene expression was detected by qRT-PCR using a 7900HT Fast Real Time PCR System thermocycler with SDS 2.3 and RQ Manager 1.2 software (Applied Biosystems, Foster City, CA, USA). Data were processed into relative gene expression levels using the 2−ΔΔCt formula, where control samples (treated with control vehicle) were used as a calibrator. All reactions were performed in triplicates.

### 4.7. Western Blot Analysis

The collected cell pellets were dissolved in 100 µL of CelLytic™ MT Cell Lysis Reagent (Sigma-Aldrich, Merck KGaA, Darmstadt, Germany) supplemented with 1 µL of Proteinase Inhibitor Cocktail (100×) (Thermo-Fischer Scientific, Waltham, MA, USA) and 10 µL of 0.2 mM PMSF (Phenylmethanesulfonyl fluoride, Sigma-Aldrich, Merck KGaA, Darmstadt, Germany). The lysates were then subjected to several fine needle aspirations for cell membrane mechanical disruption. Thereafter, the lysates were mixed on a vortex for 1 h at 4 °C, centrifuged, and the supernatant was collected as the final cell lysate. Protein concentration was evaluated by the Pierce BCA Protein Assay kit (Thermo Fischer Scientific, Waltham, MA, USA) according to the manufacturer’s protocol, and colorimetric extinction was measured at Nanodrop (Thermo Fischer Scientific, Waltham MA, USA). The lysates were reconstituted to the target volume containing 30 µg of total protein or 50 µg in the case of particular protein detection (p21) and denatured for 10 min at 95 °C with the addition of SDS Sample Loading Buffer. Electrophoresis was performed in 6–10% polyacrylamide gels, and transferred to PVDF membrane by the wet transfer method. All membranes were blocked for 1 h in 4% BSA (Bovine Serum Albumin, Sigma-Aldrich, Merck KGaA, Darmstadt, Germany) solution at room temperature, followed by overnight incubation at 4 °C with primary antibodies, i.e., SOX7 (1:500, ab94397, Abcam, Cambridge, UK), SOX17 (1:1000, ab224637, Abcam), SOX18 (1:50, sc-166025, Santa Cruz Biotechnology, Dallas, TX, USA), cyclin D1 (1:500, AFH0082, Invitrogen, Waltham, MA, USA), cyclin E (1:1000, 32-1600, Invitrogen), cyclin A1 (1:1000, MAB7046, Novus Biologicals, Centennial, CO, USA), p21 (1:1000, 2946S, Cell Signalling, Danvers, MA, USA), β-actin (1:2000, Ab8224, Abcam).

An incubation with matching secondary antibody (Cat. 711-035-150, and 711-035-152, JacksonImmunoResearch, Bar Harbor, ME, USA) at concentration of 1:5000 was performed in room temperature for 90 min. Membrane washings were performed between every step three times for 10 min by 0.1% Tween in Tris Buffered Solution. The signal was detected using Luminata Classico or Forte chemiluminescent substrate (Merck KGaA, Darmstad, Germany). The visualisation and densitometry analysis was performed with the ChemiDoc Imaging System with the ImageLab software 4.1(Bio-Rad, Marnes-la-Coquette, France). The measurements included in the statistical analysis were obtained from three independent experiments.

### 4.8. Immunohistochemistry (IHC)

Paraffin blocks containing lung tumour tissue fragments were sectioned into 5 μm thick slices and mounted on Flex IHC Microscope Slides (K8020, Dako, Glostrup, Denmark). After deparaffinisation and rehydration, the epitopes were exposed by boiling at 97 °C for 20 min using the high-pH Target Retrieval Solution in Dako PT Link (Dako, Glostrup, Denmark). Endogenous peroxidase activity was blocked by incubating the sections in En-VisionTM FLEX Peroxidase-Blocking Reagent (Dako, Glostrup, Denmark) for 5 min at room temperature. The slides were then incubated with specific primary antibodies for 20 min at room temperature. Mouse anti-SOX18 antibody (sc-166025, Santa Cruz Biotechnology) diluted 1:25 in FLEX Antibody Diluent (Dako, Glostrup, Denmark) was used to detect SOX18 expression, and mouse anti-p21 antibody (1:1000, 2946S, Cell Signaling) was used for p21 detection. After washing with EnVision FLEX Wash Buffer (Dako, Glostrup, Denmark), the slides were incubated with a secondary antibody conjugated with EnVisionTM FLEX/horseradish peroxidase (HRP; Dako, Glostrup, Denmark) for 20 min at room temperature. Subsequently, the slides were treated with a peroxidase substrate, 3,3′-diaminobenzidine (DAB), for 10 min at room temperature and counterstained with hematoxylin (EnVisionTM FLEX Hematoxylin; Dako, Glostrup, Denmark) for 7 min at room temperature. Finally, the slides were dehydrated and coverslips were mounted. All IHC reactions were performed using the Dako Autostainer Link48 (Dako, Glostrup, Denmark). The negative control was prepared without the use of the primary antibody.

### 4.9. Evaluation of IHC Reactions

The expression of p21 and SOX18 was evaluated in 48 lung tumour tissue samples by two investigators (Mateusz Olbromski and Olga Rodak) using the BX-41 light microscope at ×200 magnification (Olympus, Tokyo, Japan). Three representative spots (1.5 mm diameter) from each tumour sample were assessed. The 12-point semi-quantitative immunoreactive score (IRS) according to Remmele and Stegner was used to evaluate the intensity of the cytoplasmic reaction [63]. This method combines two variables: The percentage of positive cells (0–4) and the intensity of the colour reaction (0–3). For the nuclear reaction, a 5-point scale was used based on the percentage of positive cells: 0 points (≤1%), 1 point (2–10%), 2 points (11–25%), 3 points (25–50%), 4 points (50–75%), and 5 points (>75% of positive cells).

### 4.10. Statistical Analysis

Results underwent statistical analysis, and their graphical presentation was performed in Origin 8.1 software. Data representing normal distribution and comparable variation were evaluated by ANOVA, followed by the post-hoc Tukey test with significance set at *p*-value < 0.05. To calculate the correlation between protein expression, the Pearson correlation coefficient method was applied.

## 5. Conclusions

Previous studies have reported that SOX18 is overexpressed in various types of cancers and is associated with poor patient outcomes. Sm4, a novel compound, has been identified as a potential inhibitor of SOX18. The observed cytotoxic concentration of Sm4 in both lung cancer and normal cell lines was consistent with previous reports, indicating a similar order of magnitude of inhibitory/cytotoxic concentration values. Contrary to our initial hypothesis, the antiproliferative effect of SOX18 inhibition was not caused by upregulation of SOX7 or SOX17. Sm4-mediated cell cycle arrest showed S-phase cell accumulation, which differed among squamous cell carcinoma and lung and adenocarcinoma cell lines due to the opposing results of cyclin A1 expression. Sm4 has the potential to disrupt the cell cycle and target cancer cell growth, potentially by modulating p21 expression. Our findings suggest a complex interplay between SOX18 and p21 in the context of lung cancer, and further investigation is necessary to fully understand the relationship between these two proteins in the disease.

## Data Availability

Not applicable.

## Supplementary Materials

The following supporting information can be downloaded at: https://www.mdpi.com/article/10.3390/ijms241411316/s1.
