# Supplementary material for: Targeting SOX18 Transcription Factor Activity by Small-Molecule Inhibitor Sm4 in Non-Small Lung Cancer Cell Lines
Source: Int J Mol Sci. 2023 Jul 11;24(14):11316. doi: 10.3390/ijms241411316 (PMC10379584; doi:10.3390/ijms241411316)
Supplement: Supplementary file 1 [file ijms-24-11316-s001.zip › ijms-2425706-supplementary.pdf]

Suplemetary Information

Targeting SOX18 Transcription Factor Activity by Small-Molecule Inhibitor Sm4 in Non-Small Lung Cancer Cell Lines

Olga Rodak [1,*], Monika Mrozowska [1], Agnieszka Rusak [1], Agnieszka Gomułkiewicz [1], Aleksandra Piotrowska [1], Mateusz Olbromski [1,*], Marzenna Podhorska-Okołów [2], Maciej Ugorski [3] and Piotr Dzięgiel [1,4]

[1] Division of Histology and Embryology, Department of Human Morphology and Embryology, Wroclaw Medical University, 50-368 Wroclaw, Poland; monika.mrozowska@umw.edu.pl (M.M.); agnieszka.rusak@umw.edu.pl (A.R.); agnieszka.gomulkiewicz@umw.edu.pl (A.G.); aleksandra.piotrowska@umw.edu.pl (A.P.); piotr.dziegiel@umw.edu.pl (P.D.)
[2] Division of Ultrastructural Research, Department of Human Morphology and Embryology, Wroclaw Medical University, 50-368 Wroclaw, Poland; marzenna.podhorska-okolow@umw.edu.pl
[3] Department of Biochemistry and Molecular Biology, Faculty of Veterinary Medicine, Wroclaw University of Environmental and Life Sciences, 50-375 Wroclaw, Poland; maciej.ugorski@upwr.edu.pl
[4] Department of Physiotherapy, University School of Physical Education, 51-612 Wroclaw, Poland
* Correspondence: olga.rodak@student.umw.edu.pl (O.R.); mateusz.olbromski@umw.edu.pl (M.O.)

LIST OF CONTENTS:

1. **Figure S1**
2. **Figure S2**

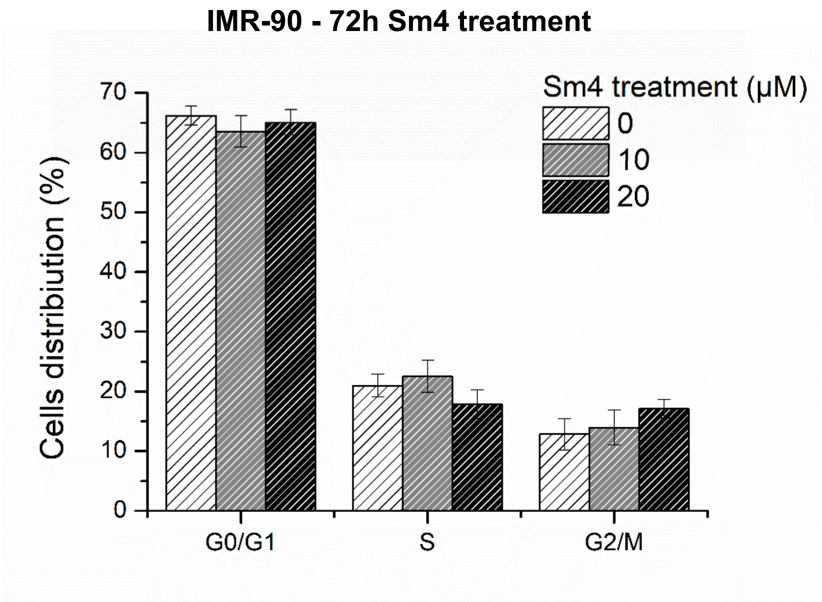

Figure S1. Cell cycle assay for IMR-90 cell line. No Statistical differences were observed in normal lung fibroblast cell line between control not treated and treated cells.

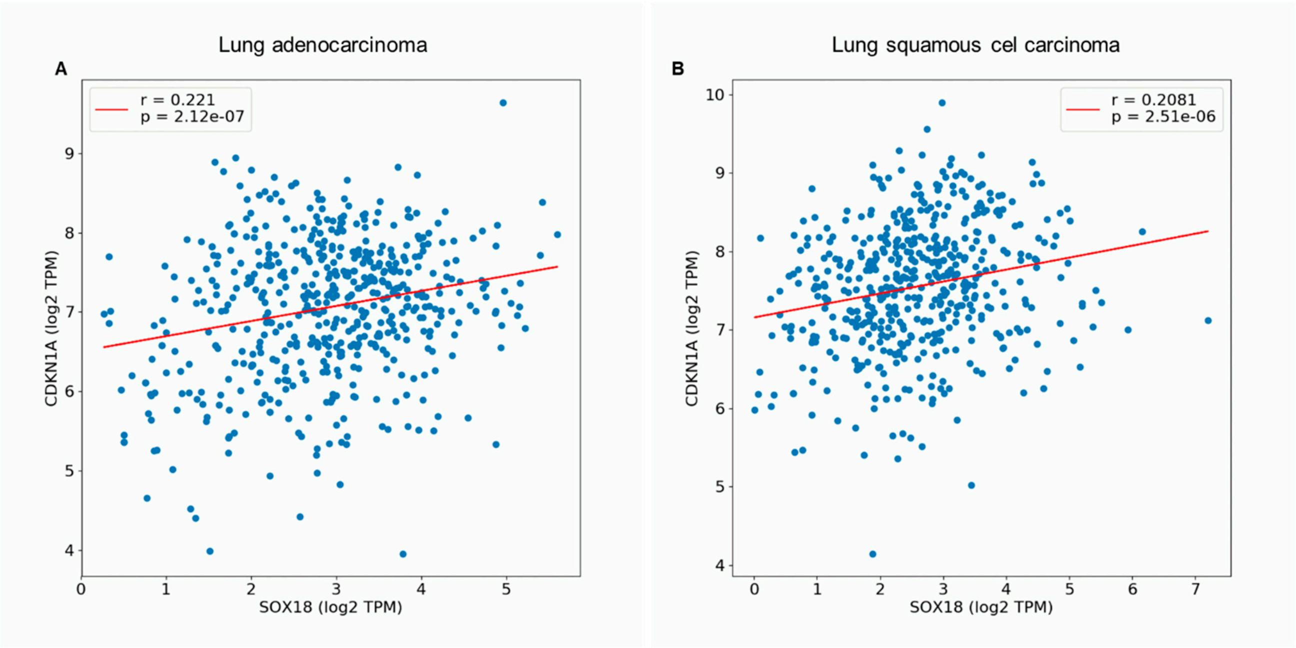

Figure S2. Correlation analysis of *SOX18* and *CDKN1A* mRNa levels. Positive correlation was obtained in both lung cancer subtype i.e.: (A) adenocarcinoma, (B) squamous cell carcinoma. The source of this data is publicly accesable OncoDb dataset (https://oncodb.org/).
